# Significance of frailty in prognosis after surgery in patients with pancreatic ductal adenocarcinoma

**DOI:** 10.1186/s12957-021-02205-6

**Published:** 2021-03-29

**Authors:** Shinichiro Yamada, Mitsuo Shimada, Yuji Morine, Satoru Imura, Tetsuya Ikemoto, Yu Saito, Katsuki Miyazaki, Takuya Tokunaga, Masaaki Nishi

**Affiliations:** Department of Surgery, Tokushima University, 3-18-15 Kuramoto-cho, Tokushima City, Tokushima, 770-8503 Japan

**Keywords:** Frailty, Pancreatic ductal adenocarcinoma, Prognostic factor

## Abstract

**Background:**

Frailty is an important consideration for older patients undergoing surgery. We aimed to investigate whether frailty could be a prognostic factor in patients with pancreatic ductal adenocarcinoma who underwent pancreatic resection.

**Methods:**

One hundred and twenty patients who underwent pancreatic resection for pancreatic ductal adenocarcinoma were enrolled. Frailty was defined as a clinical frailty scale score ≥4. Patients were divided into frailty (*n* = 29) and non-frailty (*n*=91) groups, and clinicopathological factors were compared between the two groups.

**Results:**

The frailty group showed an older age, lower serum albumin concentration, lower prognostic nutritional index, larger tumor diameter, and higher rate of lymph node metastasis than the non-frailty group (*p* < 0.05). Neutrophil–lymphocyte ratio and modified Glasgow prognostic score tended to be higher in the frailty group. Cancer-specific and disease-free survival rates were significantly poor in the frailty group (*p* < 0.05). With a multivariate analysis, frailty was an independent prognostic factor of cancer-specific survival.

**Conclusions:**

Frailty can predict the prognosis of patients with pancreatic ductal adenocarcinoma who undergo pancreatic resection.

## Background

The average life expectancy has increased all over the world. Japanese individuals showed the longest life expectancy worldwide in recent years. Treatment of cancer in older individuals has been a clinical problem owing to this increase in life expectancy [1]. Pancreatic ductal adenocarcinoma (PDAC), a type of lethal malignant tumor, has a poor prognosis, and more than half of patients are diagnosed after the age of 70 years [2]. With advances in perioperative management and surgical techniques, surgery offers a potential cure for PDAC, but surgery in older populations remains controversial [3]. Although recent studies insisted that most older patients can receive curative therapy, including surgery, older patients selected for surgery may be among the fittest and are less likely to have comorbidities [4]. In a recent meta-analysis, the overall survival of older patients with PDAC who underwent pancreatic resection was shorter compared with younger patients [5]. Thus, the best way to decide the indications of pancreatic surgery for older patients should be investigated.

Frailty is a multidimensional and heterogeneous syndrome associated with instability that can be discriminated from aging or disability [6]. Frailty is commonly assessed using summative impairment lists and algorithms based on clinical assessment [7–9]. As the number of elderly patients undergoing surgery has recently increased with developments in surgery and anesthesia, reliable methods to preoperatively assess the risks of surgery in such patients are necessary, and frailty is of great importance in predicting postoperative outcomes [9]. Although many methods can be used to assess frailty, such as the Fried frailty phenotype [10], the study of osteoporotic fracture index [11], the FRAIL scale (fatigue, resistance, ambulation, illness, loss of weight) [12], and the modified Fried index [13], few studies have compared these methods in terms of feasibility and acceptability for evaluating frailty. It was recently reported that the clinical frailty scale (CFS) was useful for predicting death or new disability after elective non-cardiac surgery [14]. CFS is a nine-point global frailty scale based on clinical evaluation in the domains of mobility, energy, physical activity, and function [15]. The CFS is reportedly a highly feasible, acceptable, and convenient instrument for clinical use in the perioperative period. We previously reported that frailty assessed using the CFS could predict the prognosis of older patients with hepatocellular carcinoma undergoing hepatic resection [1]. However, there are no reports of the CFS in patients with PDAC who underwent surgery.

Herein, we aimed to investigate whether frailty, as determined by the CFS, could be a prognostic factor in patients with PDAC undergoing pancreatic resection.

## Methods

### Patients

One hundred and twenty patients with PDAC undergoing surgery at Tokushima University Hospital from April 2006 to March 2019 were included in this retrospective study. Patients were selected using the following inclusion criteria: (a) no history of previous treatment prior to surgery and no distant metastasis and (b) pathologically proven PDAC. Patients who underwent R2 resection were excluded.

Patients’ background and preoperative characteristics, including age, sex, blood examinations, and comorbidities, were obtained from medical records. Tumor factors, including tumor markers, maximum tumor diameter, lymph node metastasis, vessel invasion, differentiation, and tumor stage according to Japan Pancreas Society 7th edition guidelines [16] were also collected. Immunonutritional factors were also measured using total lymphocyte count (TLC), neutrophil–lymphocyte ratio (NLR) [17], prognostic nutritional index (PNI) [18], and modified Glasgow prognostic score (mGPS) [19]. Assessment of the CFS was performed in accordance with our previous study [1]. Frailty was defined as a CFS score of ≥4. Patients were divided into a frailty group (*n* = 29) and a non-frailty group (*n* = 91). The study was approved by the institutional review board of Tokushima University (No. 3786).

### Statistical analysis

The unpaired Mann–Whitney *U*-test or *χ*^2^ test was used to compare clinicopathological factors between the two groups. Cancer-specific and disease-free survival rates were calculated by the Kaplan–Meier method, and differences were compared using the log-rank test. A multivariate analysis was performed using the Cox proportional hazards regression model. For all statistical analyses, a *p* value of <0.05 was considered statistically significant. All statistical analyses were performed using the JMP 8.0.1 statistical software (SAS Campus Drive, Cary, NC, USA).

## Results

### Clinicopathological factors

Table 1 shows the clinicopathological factors of patients in the frailty and non-frailty groups. The frailty group had an older age (*p* < 0.05) and a higher rate of pulmonary dysfunction (*p* = 0.07). Regarding immunonutritional status, NLR and mGPS tended to be higher in the frailty group compared with the non-frailty group (*p* = 0.09 and *p* = 0.12, respectively). However, there was no significant difference between the two groups. PNI and serum albumin concentration were significantly lower in the frailty group compared with the non-frailty group (*p* < 0.05). Regarding tumor factors, the frailty group showed a significantly larger tumor diameter and a higher rate of lymph node metastasis compared with the non-frailty group (*p* < 0.05).

**Table 1:** Patients’ characteristics according to frailty status

Parameters	No frailty (*n*=91)	Frailty (*n*=29)	*P*-value
Preoperative variable			
Age (years)	68.0 ± 8.8	75.6 ± 6.8	<0.01
Sex (male/female)	38/26	11/6	0.69
Diabetes (+/−)	41/49	17/12	0.20
Pulmonary dysfunction (+/−)	19/72	9/20	0.07
Hemoglobin (g/dL)	13.1 ± 1.6	12.8 ± 1.6	0.55
AST (U/L)	50.4 ± 71.7	39.1 ± 35.5	0.83
PT (s)	12.0 ± 1.4	12.0 ± 1.3	0.66
Immuno-nutritional status			
TLC (/μL)	1467 ± 513	1350 ± 524	0.22
NLR	2.8 ± 1.5	3.5 ± 2.0	0.09
PNI	47.3 ± 5.0	43.1 ± 7.8	<0.01
Albumin (g/mL)	4.0 ± 0.5	3.6 ± 0.7	0.01
CRP (mg/dL)	0.4 ± 0.8	0.9 ± 2.4	0.40
mGPS (0/1, 2)	70/21	18/11	0.12
Tumor factors			
CEA (ng/mL)	4.1 ± 10.1	3.5 ± 4.7	0.87
CA19-9 (U/mL)	388 ± 713	1038 ± 2871	0.11
Maximum diameter (cm)	2.8 ± 1.1	3.3 ± 1.4	0.03
Lymph node metastasis (+/−)	65/26	15/14	0.04
Lymphatic invasion (+/−)	32/59	6/23	0.12
Venous invasion (+/−)	22/69	4/25	0.21
Differentiation (well, mod/por)	79/12	23/6	0.27
Stage (I / II)	27 / 64	4/25	0.07
Perioperative status			
Procedure (PD/DP/TP)	58/32/1	20/7/2	0.18
Operative time (min)	396 ± 113	444 ± 120	0.02
Bleeding (ml)	264 ± 245	319 ± 264	0.35
Postoperative complication	42 (46%)	14 (48%)	0.84
Postoperative hospital stay (days)	32 ± 22	31 ± 16	0.89

*AST* aspartate aminotransferase, *PT* prothrombin time, *TLC* total lymphocyte count *NLR* neutrophil–lymphocyte ratio, *PNI* prognostic nutritional index, *CRP* C-reactive protein, *mGPS* modified Glasgow Prognostic Score, *CEA* carcinoembryonic antigen, *CA19-9* carbohydrate antigen 19-9, *PD* pancreatoduodenectomy, *DP* distal pancreatectomy, *TP* total pancreatectomy

Data are expressed as the mean ± SD

### Postoperative features

Operative time was significantly longer in the frailty group compared with the non-frailty group (*p* < 0.05). There was no significant difference in operative procedure, volume of blood loss, postoperative complications, or length of postoperative hospital stay between the two groups.

### Cancer-specific and disease-free survival rates

**Fig. 1:**
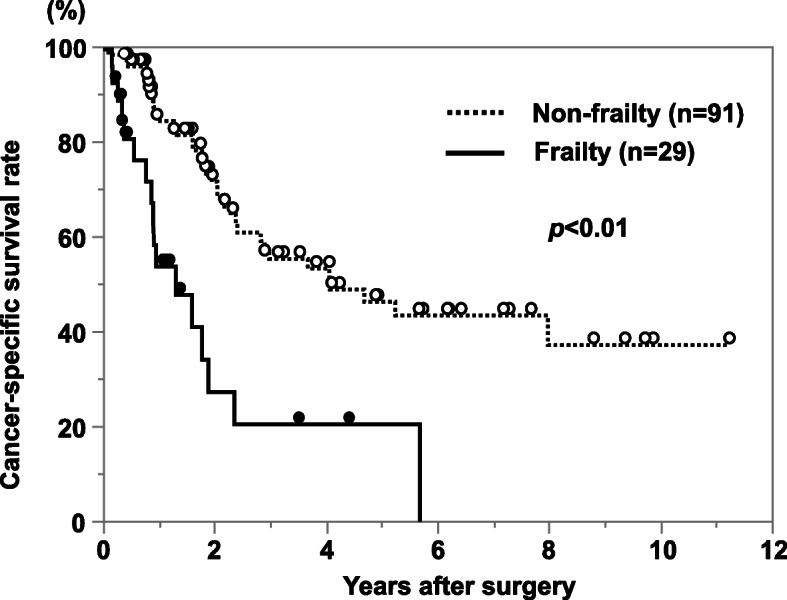
Comparison of postoperative cancer-specific survival rate of patients with PDAC according to frailty status. The frailty group has a significantly worse prognosis than the no frailty group (*p*<0.01)

**Fig. 2:**
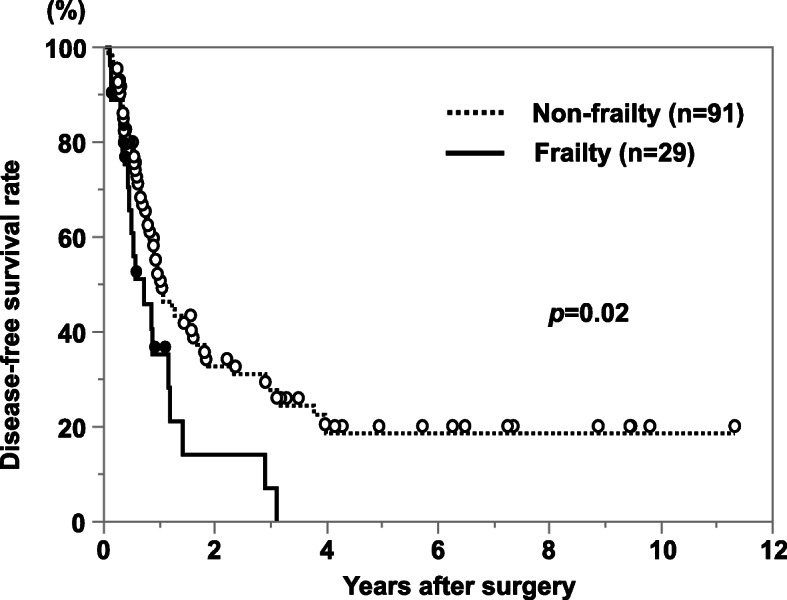
Comparison of postoperative disease-free survival rate of patients with PDAC according to frailty status. The frailty group has a significantly worse prognosis than the no frailty group (*p*=0.02)

**Fig. 3:**
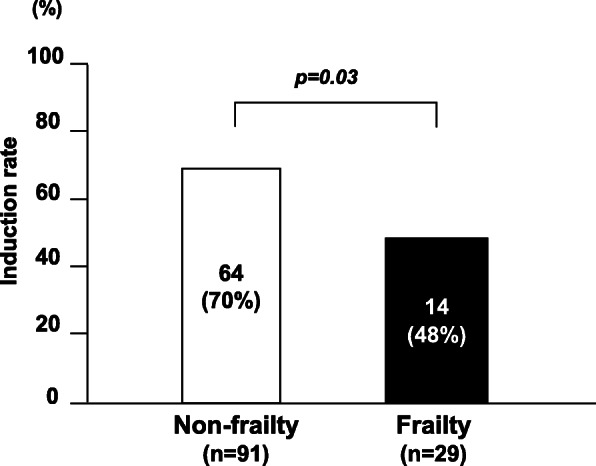
Comparison of induction rate of adjuvant chemotherapy according to frailty status. The frailty group has a significantly lower induction rate than the no frailty group (*p*=0.03)

**Fig. 4:**
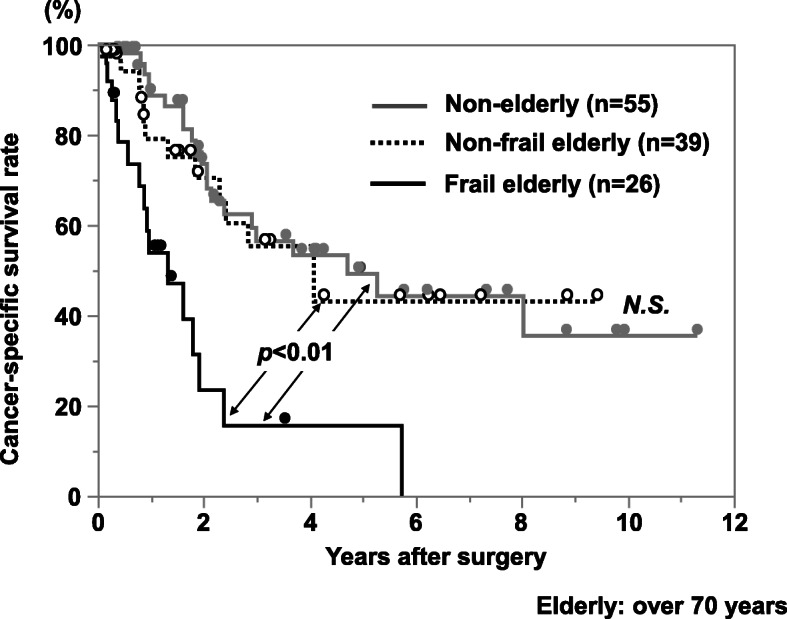
Comparison of overall postoperative cancer-specific survival rate of patients with PDAC according to frailty and aging status. The frail elderly group showed a significantly worse prognosis than the other two group, no elderly and no frail elderly groups (*p*<0.01). The no frail elderly group showed comparable outcome compared with the no elderly group

**Fig. 5:**
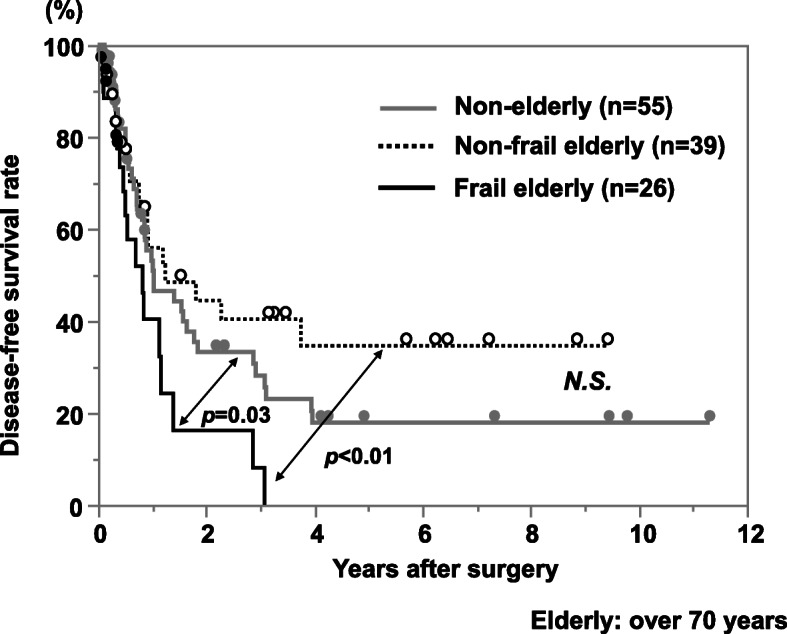
Comparison of postoperative disease-free survival rate of patients with PDAC according to frailty and aging status. The frail elderly group showed a significantly worse prognosis than the other two group, the no elderly and the no frail elderly groups (*p*<0.01). The no frail elderly group showed comparable outcome compared with the no elderly group

The cancer-specific survival rate of patients with PDAC after surgery was significantly lower in the frailty group compared with the non-frailty group (*p* < 0.01; Fig. 1). Cancer-specific 3-year survival rates in the frailty and non-frailty groups were 20.1% and 55.7%, respectively. With a univariate analysis of cancer-specific survival, an age of >70 years, a tumor size of ≥3 cm, lymph node metastasis, advanced tumor stage, lymphatic invasion, a cancer antigen (CA) 19-9 concentration of ≥37 U/ml, frailty, an mGPS of 1 or 2, a PNI of <40, and R1 resection were prognostic factors. A multivariate analysis showed that lymphatic invasion, a CA 19-9 concentration of ≥37 U/ml, frailty, and an mGPS of 1 or 2 were independent prognostic factors (Table 2). In terms of disease-free survival, the frailty group showed a significantly poor prognosis compared with the non-frailty group (*p* = 0.02; Fig. 2). With a univariate analysis, a tumor size of ≥3 cm, lymph node metastasis, advanced tumor stage, poorly differentiated tumor, lymphatic invasion, a CA 19-9 concentration of ≥37 U/ml, frailty, and an mGPS of 1 or 2 were prognostic factors. A multivariate analysis showed that poorly differentiated tumor and a CA 19-9 concentration of ≥37 U/ml were independent prognostic factors (Table 3). In terms of adjuvant chemotherapy, the induction rate was significantly lower in the frailty group compared with the non-frailty group (48% vs. 70%, respectively; *p* < 0.05; Fig. 3). When patients were divided into the groups (non-elderly [<70 years, *n* = 55], non-frail elderly [≥70 years, *n* = 39], and frail elderly [*n* = 26]), there was no significant difference in tumor factors and immune-nutrition status between the non-elderly group and the non-frail elderly group. There was also no significant difference in cancer-specific survival and disease-free survival between these two groups. The frail elderly group showed significantly worse survival compared with the other two groups (*p* < 0.05; Figs. 4 and 5).

**Table 2:** Results of univariate and multivariate analysis for cancer-specific survival

Variable	Three-year survival rate (%)	Univariate	Multivariate	
		*P*-value	HR (95% CI)	*P*-value
Age (<70/≥70 years)	56.6/39.4	0.04	1.54 (0.67–3.41)	0.30
Sex (M/F)	42.8/53.6	0.45		
Tumor diameter (<3cm/≥3cm)	61.0/31.0	<0.01	1.47 (0.71–3.12)	0.30
Lymph node metastasis (+/−)	35.2/54.5	0.03	1.29 (0.64–2.60)	0.47
Stage (I/II)	78.5/34.3	<0.01	1.84 (0.71–5.20)	0.21
Differentiation (well, mod/por)	50.2/36.7	0.08		
Lymphatic invasion (+/−)	37.9/64.8	0.01	3.66 (1.56–9.49)	<0.01
Venous invasion (+/−)	44.0/58.0	0.12		
CEA (<5/≥5 ng/mL)	49.3/33.9	0.48		
CA19-9 (<37/≥37 U/mL)	74.7/35.0	<0.01	3.39 (1.43–9.29)	<0.01
Frailty (+/−)	20.1/55.7	<0.01	2.94 (1.36–6.40)	<0.01
mGPS (0/1, 2)	60.1/14.8	<0.01	2.36 (1.08–5.10)	0.03
PNI (<40/≥40)	66.7/89.4	0.34		
R0/R1	53.8/27.9	0.02	2.06 (0.94–4.45)	0.07

**Table 3:** Results of univariate and multivariate analysis for disease-free survival

Variable	Three-year survival rate (%)	Univariate	Multivariate	
		*P*-value	HR (95% CI)	*P*-value
Age (<70/≥70 years)	28.9/29.0	0.92		
Sex (M/F)	30.0/27.7	0.80		
Tumor diameter (<3cm/≥3cm)	40.1/15.0	<0.01	0.98 (0.55–1.75)	0.95
Lymph node metastasis (+/−)	16.1/36.4	0.04	1.00 (0.56–1.79)	0.99
Stage (I/II)	46.8/21.4	<0.01	1.71 (0.88–3.41)	0.11
Differentiation (well, mod/por)	31.8/14.8	<0.01	2.71 (1.35–5.16)	<0.01
Lymphatic invasion (+/−)	23.3/42.5	0.04	1.14 (0.64–2.07)	0.65
Venous invasion (+/−)	25.5/42.7	0.06		
CEA (<5 / ≥5 ng/mL)	30.0/18.1	0.06		
CA19-9 (<37/≥37 U/mL)	47.2/19.5	<0.01	2.17 (1.16–4.29)	0.01
Frailty (+/−)	13.1/32.6	0.02	1.48 (0.83–2.55)	0.18
mGPS (0/1, 2)	32.9/19.3	0.01	1.75 (0.94–3.20)	0.08
PNI (<40/≥40)	26.1/29.7	0.09		
R0/R1	29.1/23.6	0.16

## Discussion

In the present study, the relationship between clinicopathological factors and frailty was investigated in patients with PDAC and the usefulness of frailty as a prognostic factor. Frailty showed correlation with (1) a low serum albumin concentration and PNI, (2) a high NLR and mGPS, (3) a large tumor diameter and a high rate of lymph node metastasis, and (4) worse cancer-specific and disease-free survival. Furthermore, aging itself was not an independent prognostic factor for survival. To our knowledge, the present study is the first to use the CFS to show a significant association between frailty and post-surgical prognosis in patients with PDAC.

Frailty is an aggregate expression of susceptibility to adverse health outcomes because of age- and disease-related deficits that accumulate across multiple domains [15]. Some reports in geriatric patients show that frailty correlates with functional decline, hospitalization, and death [9, 14]. In our study, the CFS was used to assess frailty. Many methods can be used to assess frailty [10–13]; however, these methods require multiple questionnaires. The CFS is less quantitative compared with other methods that use clinical questionnaires, but the CFS correlates with other established assessment methods [7]. Furthermore, the CFS can easily assess the general appearance and frailty of patients at the first check-up.

Recently, it has emerged that frailty is associated with cancer-specific survival in patients with some malignancies [1]. In our study, frailty correlated with poor survival rate after surgery and advanced tumor state, such as larger tumor diameter and higher rate of lymph node metastasis. Although the mechanism by which frailty influences cancer malignancy and recurrence has not yet been determined, frailty is associated with inflammatory markers, such as a high mGPS. mGPS was reported to represent the presence of inflammatory response and correlated with decreased muscle mass, lower functional level, and inflammatory and angiogenic cytokines [20]. It has also been reported that patients with frailty and various solid malignancies show a high mGPS, a more advanced tumor stage, and a poor prognosis [21]. Hypoalbuminemia, which mainly causes a high mGPS, partially reflect an immunosuppressed status and weak systemic defense; thus, it may be related with poor survival outcomes [22]. This can affect overall and disease-free survival in patients with various cancers. In the present study, the frailty group showed significantly lower albumin level and tendency of higher mGPS compared with the non-frailty group. Furthermore, longitudinal aging studies of Singapore [23] showed that frailty was associated with low ɤ/δ T cells and exhausted B cell. These findings, which indicate systemic inflammation and immunosuppression, might be related to tumor progression such as large tumor diameter and higher rate of lymph node metastasis, and poor survival in our study.

Although frailty was an independent prognostic factor for cancer-specific survival, it was not a prognostic factor for disease-free survival. One reason for this discrepancy is the induction rate of adjuvant chemotherapy. The frailty group showed a significantly lower rate of induction compared with the non-frailty group. In pancreatic cancer, induction of adjuvant chemotherapy is a prognostic factor [24]. Regarding the relationship between frailty and chemotherapy, frailty is associated with a low adjuvant chemotherapy induction rate in patients with stage III colon cancer [25]. In our study, a low induction rate of adjuvant chemotherapy may have led to worse cancer-specific survival.

In this study, aging itself was not an independent prognostic factor, and the non-frail elderly group showed comparable outcomes compared with the non-elderly group. Recently, the number of reports insisting that pancreatic resection of PDAC can be performed safely on older patients with acceptable risks is increasing [26]. However, frailty is a prognostic factor in several cancers [1], as shown in the present study. For older patients with frailty, preoperative rehabilitation improves postoperative motor function, quality of life, and possibly surgical outcomes. Perioperative intervention seems important during pancreatic resection for postoperative outcomes and good induction of adjuvant chemotherapy.

The present study has several limitations. First, it was a single-center study, and the study cohort was relatively small. Larger prospective studies are necessary to confirm our findings. Second, we only used CFS scores to assess frailty in this study. In the future, we plan to assess other variables associated with frailty and cancer, such as sarcopenia and dynapenia.

## Conclusion

Frailty can predict the prognosis of patients with PDAC undergoing pancreatic resection. Elderly patients without frailty showed comparable outcome with non-elderly patients.

## Data Availability

Not applicable
